# Oxygen Transfer Characteristics of Miniaturized Bioreactor Systems

**DOI:** 10.1002/bit.24824

**Published:** 2013-01-17

**Authors:** Timothy V Kirk, Nicolas Szita

**Affiliations:** Department of Biochemical Engineering, University College LondonTorrington Place, London, WC1E 7JE United Kingdom

**Keywords:** minibioreactors, microbioreactors, oxygen transfer, oxygen monitoring

## Abstract

Since their introduction in 2001 miniaturized bioreactor systems have made great advances in function and performance. In this article the dissolved oxygen (DO) transfer performance of submilliliter microbioreactors, and 1–10 mL minibioreactors was examined. Microbioreactors have reached *k*_L_*a* values of 460 h^-1^, and are offering instrumentation and some functionality comparable to production systems, but at high throughput screening volumes. Minibioreactors, aside from one 1,440 h^-1^ *k*_L_*a* system, have not offered as high rates of DO transfer, but have demonstrated superior integration with automated fluid handling systems. Microbioreactors have been typically limited to studies with *E. coli*, while minibioreactors have offered greater versatility in this regard. Further, mathematical relationships confirming the applicability of *k*_L_*a* measurements across all scales have been derived, and alternatives to fluorescence lifetime DO sensors have been evaluated. Finally, the influence on reactor performance of oxygen uptake rate (OUR), and the possibility of its real-time measurement have been explored. Biotechnol. Bioeng. 2013; 110: 1005–1019. © 2012 Wiley Periodicals, Inc.

## Introduction

Performance of bioreactors is typically qualified by consideration of transport process and biochemical conversion (Lübbert and Bay Jørgensen, 2001). Oxygen transfer to the liquid phase is perhaps the most important transport process, due to both the relatively low solubility of oxygen in water and the high demand from aerobic bioprocesses. Indeed, in many such processes, for every unit of biomass produced approximately an equal mass of oxygen must be consumed (Lübbert and Bay Jørgensen, 2001).

Accordingly, monitoring of dissolved oxygen (DO) concentration is of particular importance at all scales, and characterization of transfer is a fundamental concern when scaling down to mini and microvolumes. The most commonly presented characterization of bioreactor DO transfer is the oxygen volumetric mass transfer coefficient, *k*_L_*a*. Assuming applicability across scales, a single number can be used to compare DO transport performance from microliter to production volume.

In optimizing bioprocesses as much data from as many process variables as practically available is desired (Harms et al., 2002). Cell density and pH are the most obvious of these parameters; cell density being so fundamental that monitoring is considered to be indispensable. Consequently, we have limited the definition of bioreactors to those that offer monitoring of DO and cell density at a minimum. Commercially available systems were excluded when lacking peer reviewed analysis.

**Figure 1:**
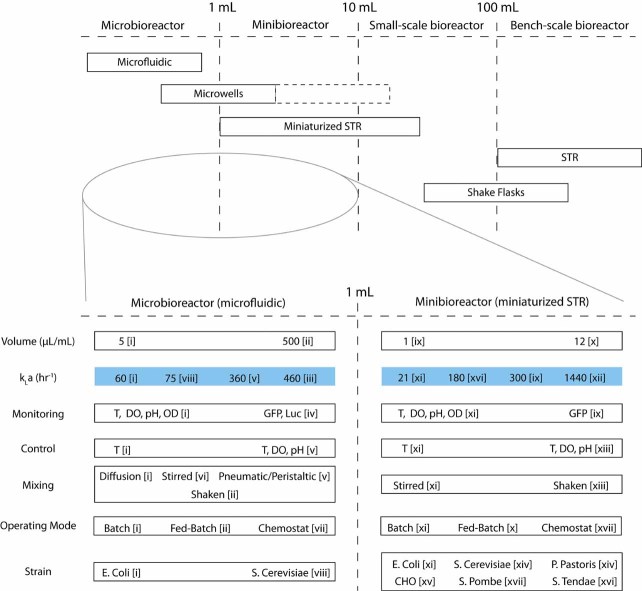
Classification of bioreactors and cell culture systems by volume. Property ranges are illustrative of the range of function. In the cases of monitoring and control, ranges give the typical capabilities of systems in the field and the more advanced capabilities demonstrated. Where appropriate the 1st published account of a system with the properties described is referenced. For the quoted *k*_L_*a* values, references are given with the mixing mechanism used in each system, in order to relate this parameter to a key design characteristic. Key: i (Zanzotto et al., 2004), diffusion; ii (Funke et al., 2010b); iii (Funke et al., 2010a), shaken; iv (Zanzotto et al., 2006); v (Lee et al., 2006), peristaltic deflection of gas-permeable membrane; vi (Szita et al., 2005); vii (Zhang et al., 2006a); viii (Zhang et al., 2006b), stirred with magnetic bar; ix (Harms et al., 2006), stirred with impeller and sparged; x (Weuster-Botz et al., 2005); xi (Kostov et al., 2001), stirred with magnetic bar and sparged; xii (Puskeiler et al., 2005), stirred with impeller and sparged; xiii (Tang et al., 2006); xiv (Isett et al., 2007); xv (Chen et al., 2009); xvi (Hortsch et al., 2010), stirred with impeller; xvii (Klein et al., 2012).

Since 2001, when Kostov et al. (2001) integrated optical sensors in a stirred and sparged cuvette, several small systems have been developed to extended bioreactor functions and instrumentation to scales suitable for high throughput screening and process development. These fall into two classes—submilliliter microbioreactors (Lee et al., 2006; Schäpper et al., 2010; Szita et al., 2005; Zanzotto et al., 2004; Zhang et al., 2006a, b), and 1–10 mL minibioreactors (Kostov et al., 2001; Lamping et al., 2003; Puskeiler et al., 2005; Tang et al., 2006; Weuster-Botz et al., 2005). Ten milliliters was chosen as an upper limit on volume to make a clear distinction between bench scale and miniaturized bioreactors. To place these small systems in perspective, their volume scales and properties were illustrated in Figure 1.

As the field has developed, *k*_L_*a* values have increased to >360 h^-1^ for microbioreactors (Funke et al., 2010a; Lee et al., 2006) and >1,000 h^-1^ for minibioreactors (Puskeiler et al., 2005), allowing analogy with culture systems increasing in scale from microwell plates, through shaker flasks, various stirred tank reactors (STRs), and up to production scale bioreactors. Typical *k*_L_*a* values for these systems are given in Table I. The oxygen transfer properties of these systems have been extensively characterized and reviewed in the past. Kumar et al. (2004), Duetz (2007), Micheletti and Lye (2006), Betts and Baganz (2006), and Islam et al. (2008) are recommended for microwell plates; Maier and Büchs (2001), Maier et al. (2004) and Büchs et al. (2000a, b, 2001) for shaker flasks; and Junker (2004) for pilot scale STRs. Additionally, Gill et al. (2008) characterized a typical small scale (100 mL) bioreactor.

**Table I:** Typical DO transfer properties of various scale culture systems from process development to production.

Class	Volume range	*k*_L_*a* range (h^-1^)	Refs.
Microwell plates (384)	20–125 µL	Up to 140	Duetz and Witholt (2004)
Microwell plates (96)	100 µL to 2 mL	20–250	Islam et al. (2008)
Shaker flasks	25 mL to 2 L	10–180	Maier and Büchs (2001)
Bench-scale STR	100 mL to 1 L	Up to 400	Gill et al. (2008)
	1–30 L	60–360	Özbek and Gayik (2001)
Pilot scale STR	30–2,000 L	170–740	Junker (2004)
	14,000–20,000 L	340–380	Junker (2004)
Production scale STR	>20,000 L	Up to 1,800	Nielsen et al. (2003)

*k*_L_*a* values were chosen to reflect the ranges generally reported in the literature for culture of microbes.

Higher values may be possible, especially for bench-scale STRs. References were chosen that either measured *k*_L_*a* directly or offered a comprehensive review. Production scale systems may be more likely to offer the *k*_L_*a* range reported by Junker (2004) for large pilot plant STRs, with very high *k*_L_*a*’s only available in exceptional circumstances (Charles and Wilson, 2002).

As *k*_L_*a* values of ∼400 h^-1^ (Junker, 2004), and as high as 1,800 h^-1^ (Nielsen et al., 2003), are available at production scale, much emphasis must still be placed on maximizing oxygen transfer in miniaturized bioreactors. However, as production bioreactors vary across a great range of *k*_L_*a* values, the ability to vary the miniaturized system's performance to match them may be desired.

To adequately perform in a bioreactor environment a DO sensor must offer accurate and stable performance over the required range of DO concentrations for the duration of the experiment. Temporal response should be sufficiently fast for characterization of transient phenomena, such as DO concentration changes during *k*_L_*a* measurements. As *k*_L_*a* values of ∼360 h^-1^ (0.1 s^-1^) (Lee et al., 2006) have been demonstrated, response times should be on the order of seconds. Sensors must be sterilizable, stable over the foreseen range of fluidic conditions, and suitable for integration in reactors and with other sensors. The latter requirement is not trivial, and infers that the sensor should not significantly interfere with the culture medium—it should not leach material into it, and particularly pertinent for a small volume, nor should it consume sufficient oxygen to skew the measurement. For high throughput processing or screening sensors must also be suitable for multiplexing. These requirements have limited consideration to the now commercially established fluorescence lifetime based optical sensors, and miniaturizations of the Clarke type electrodes that still dominate in conventional larger scale systems.

This review examines and analyses DO monitoring and control, transfer performance, and characterization techniques and models in published examples of mini and microbioreactors, and how their designs produce these characteristics, limit their function, and place them amongst the hierarchy of process optimization systems. Finally, methods for expanding analysis of the data collected from such systems via real-time oxygen uptake rate (OUR) calculation are briefly examined.

## DO Measurement and Instrumentation

Traditionally, DO concentration in bioreactors has been measured amperometrically with Clark-type electrodes (Bambot et al., 1994). This electrochemical system operates (nowadays) with three electrodes typically—a cathode (working electrode) where oxygen is reduced, an anode (counter electrode) where electrons are supplied, and a reference electrode to set the bias of the system. Four electrons are exchanged per oxygen molecule consumed, implying a linear relationship between signal and analyte concentration. However, this also implies that signal magnitude may be flow dependent, as local consumption of DO will lead to boundary layer phenomena, where availability of DO at the electrode may be constrained by mass transfer. Additionally, this consumption may make Clark-type electrodes unsuitable for use in miniaturized devices due to consumption of the relatively low quantity of DO available. Indeed, Lee and Tsao (1979) noted that they are unsuitable for measuring low DO concentrations in dense microbial cultures; a limitation that applies even in lab scale systems.

**Figure 2:**
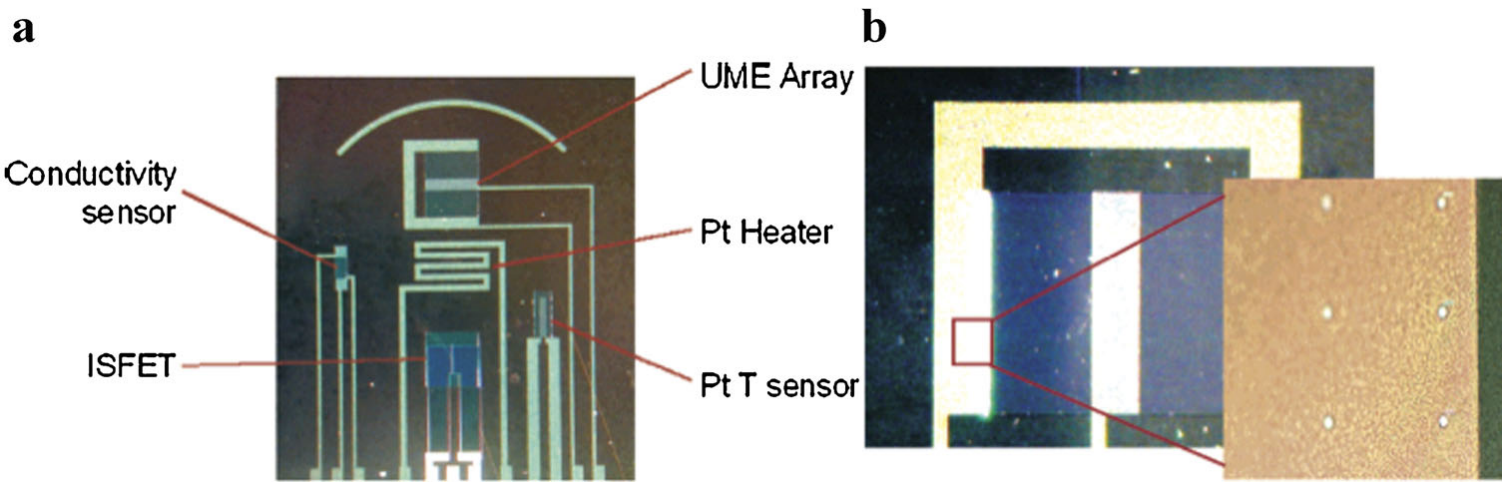
a: Miniaturized array of sensors for measuring DO concentration (UMEA), pH (ISFET), and cell density (conductivity sensor). b: UMEA electrodes, showing individual electrodes formed by photo patterning polyimide to give 114 µm × 4 µm diameter apertures spaced 50 µm apart. UMEA electrodes were previously fabricated by sputtering platinum (with a tantalum adhesion layer) on an oxidized silicon substrate to give two 200 µm × 1,000 µm electrodes. The device shown recorded currents of ∼5 to 30 nA over the nitrogen to air range of DO, which corresponds to ∼0.05 to 0.3 nmol/h oxygen consumption. At 50% DO in a 50 µL reactor with *k*_L_*a* of 100 h^-1^, this corresponds to 0.03% of the oxygen transfer rate (OTR). In later work, they were dissatisfied with the signal strength of the UMEA, leading them to operate it as a single electrode (Krommenhoek et al., 2008). This increased maximum current to ∼700 nA. Any effect on mass transfer dependence was not published. At 50% air DO, consumption of oxygen corresponds to ∼1% OTR. Problems with foaming agent addition causing changes in signal magnitude were also reported. Response time was not reported. Figure is reproduced with permission from Krommenhoek et al. (2007) (Copyright 2007 American Chemical Society).

To date, reference electrodes have proven difficult to miniaturize, hindering integration at the microscale. To alleviate this and other drawbacks, Krommenhoek et al. (2007) moved the reference electrode off chip, and pursued miniaturization of on chip electrodes below 5 µm in diameter—so called “ultra miniaturization” (UM) (Fig. 2). It had been reported that at this dimension the equilibrium DO profile is developed rapidly, and convective mass transfer is of little influence (Brett and Brett, 1993). This was demonstrated for a sensor array in a bench-scale system by showing independence of signal to agitation speed (Krommenhoek et al., 2007). To improve signal to noise ratio multiple UM electrodes have been used in an array, where they are sufficiently separated to avoid interfering with each other's DO concentration boundary layer development (Krommenhoek et al., 2007).

The low currents through the sensor corresponded to 0.03% of the oxygen transfer rate (OTR) into a 50 µL reactor with *k*_L_*a* of 100 h^-1^ at 50% DO. In later work, dissatisfied with the signal strength of the ultra microelectrode array (UMEA), they operated it as a single electrode (Krommenhoek et al., 2008). No effect of the larger electrode on mass transfer dependence was published. DO consumption increased to ∼1% OTR. Problems with foaming agent addition causing changes in signal magnitude were also reported. Response time was not reported.

Attempts to use the system in microscale reactors proved less successful however. In Krommenhoek's PhD thesis two 100 µL scale reactors were fabricated from microwells, with magnetic stir bars for mixing (Krommenhoek, 2007). They reported problems with sensor cross talk, preventing simultaneous sensor measurement, and serious signal decline over the 8-h fermentation duration, which was attributed to fouling of UMEA, possibly blocking the electrodes. This indicates that shear dependence was not alleviated.

**Figure 3:**
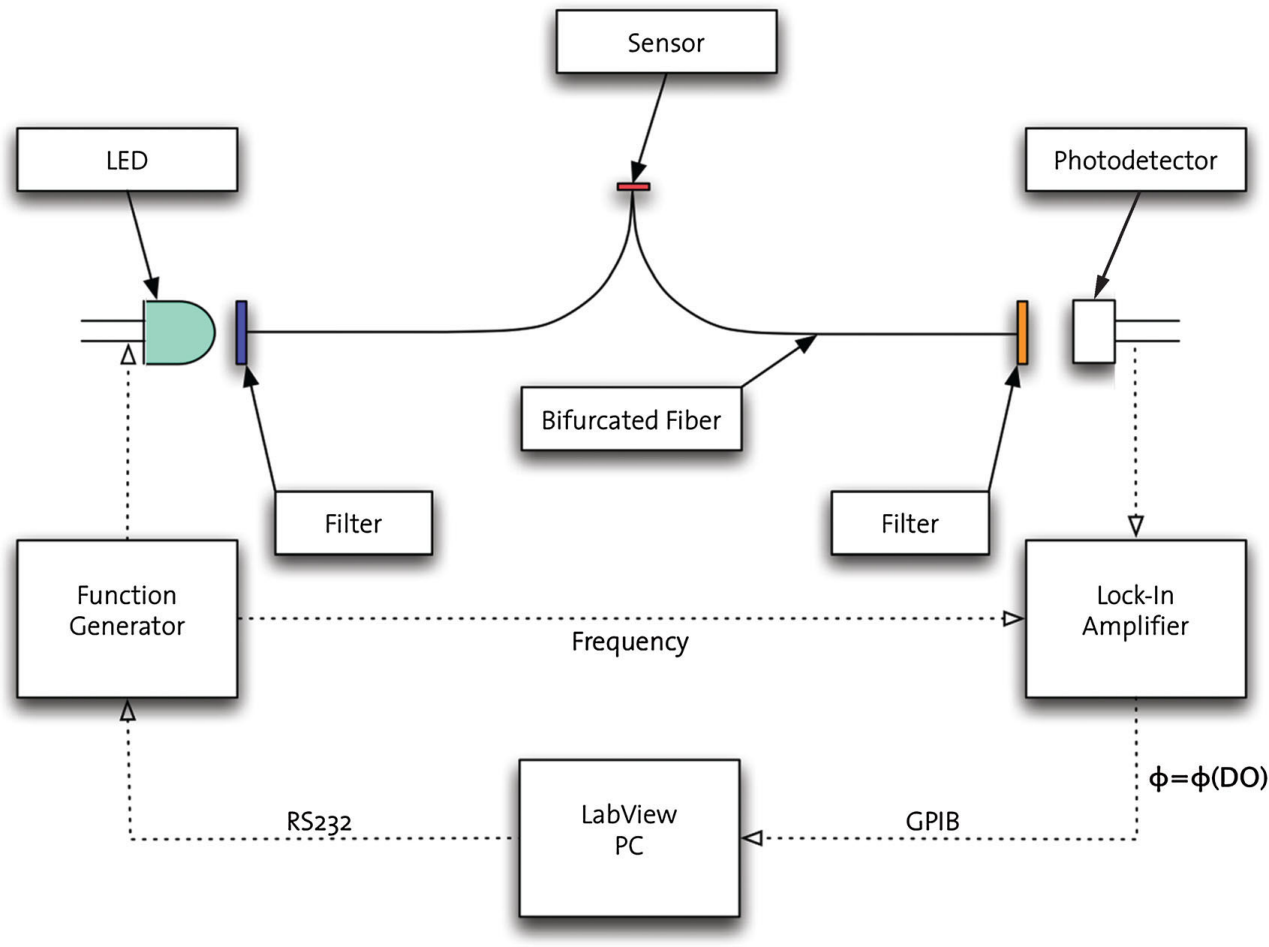
Schematic of lock-in amplifier based apparatus for fluorescence lifetime DO measurement used in Zanzotto et al. (2004). Optoelectronic components may differ in commercial systems. Sensor spots are interrogated via bifurcated fiber with a sinusoidal signal. Duschinsky (1933), and others subsequently using modern quantum mechanical methods (Armstrong and Feneuille, 1975), showed that sinusoidal excitation of a fluorophore results in sinusoidal emission at the same frequency, but with a phase shift and different amplitude. This phase shift is measured by the lock in amplifier. The phase shift is a function of decay time (which is a function of oxygen concentration) and excitation frequency, described by the equation tan(*θ*) = 2*πfτ*, where *θ* is the phase shift, *τ* the fluorescence decay time, and *f* the excitation frequency. Accordingly, phase shift will change with fluorescence decay time, and hence oxygen concentration, predicting a decreasing phase shift with increasing oxygen concentration, as there will be relatively less fluorophore molecules able to fluoresce (Shives et al., 2002). The phase shift should not significantly change with photo-bleaching, as only the ratio of fluorescing to non-fluorescing molecules determines decay time. The quenching does impose some limitations though. Reduced fluorescence intensity reduces signal to noise at higher concentrations of DO, and the shape of the Stern–Volmer curve predicts reduced sensitivity with rising DO.

Bacon and Demas (1987) presented an oxygen quenched, ruthenium based transition metal–organic complex fluorescent dye immobilized in silicone suitable for integration with a cuvette (Bacon and Demas, 1987). By measuring fluorescence lifetime they were able to non-invasively determine oxygen partial pressure optically, with high repeatability and rapid response, over the range corresponding to air. The oxygen concentration is then obtained from the partial pressure by Henry's law (Wolfbeis, 1991, p. 20). A schematic of apparatus used for fluorescence lifetime measurement of DO in Zanzotto et al. (2004) is illustrated in Figure 3. Implementations of the lifetime measurement vary (Schneider et al., 2010), and optoelectronic components may differ in commercial systems.

Improvements proliferated (Demas and DeGraff, 1991), and Bambot et al. (1994) demonstrated a planar silicone patch (500 µm thick × 5 mm diameter), containing a similar fluorophore, suitable for integration with optical fibers or microbioreactors. Since then a number of commercial products have appeared, with improvements in size, stability, and sensitivity. Common to all products have been the fundamental advantages of these sensors: non-invasive measurement of DO concentrations, no oxygen consumption, ease of integration in small volumes, and the robustness of fluorescence lifetime measurement. Indeed, from Kostov et al. (2001) to Lee et al. (2011) all published micro- and minibioreactor studies with DO monitoring have used such sensors. Similarly, pH sensors in these studies have been fluorescence lifetime based as well. Unlike their DO counterparts however, their operation is limited to a subset of typical process conditions (Hortsch et al., 2010).

DO sensors should be easy to integrate and be suitable for multiplexing. When multiplexed, multiple sensors can be measured with a single amplifier or data acquisition system. To maximize utility of bioreactor arrays, monitoring of all their individual bioreactors is desired. Optical sensors have typically been secured in bioreactors with adhesive (Szita et al., 2005; Weuster-Botz et al., 2005; Zanzotto et al., 2004; Zhang et al., 2006a, b) or formed in situ (Lee et al., 2006), and interrogated non-invasively with optical fibers (Lee et al., 2006; Szita et al., 2005; Zanzotto et al., 2004; Zhang et al., 2006a, b). Multiplexing of DO measurement has been achieved in several ways, though none involve optical multiplexing, and all but one has used individual photodetectors and LED light sources for each sensor (Szita et al., 2005). Commercial sensor response times are typically reported on the order of 5 s (Zanzotto et al., 2004). Furthering the case for these sensors, Hanson et al. (2007) demonstrated a high degree of correlation with industry standard electrochemical DO monitoring during fermentations in bench-scale bioreactors.

Weuster-Botz et al. (2005), Tang et al. (2006), Chen et al. (2009), and Isett et al. (2007) mounted reactors on commercial 24-well plate format measurement blocks. Lee et al. (2006) directly addressed each reactor's sensor with fibers, and Szita et al. (2005) used a stepper motor to move a fiber bundle between reactors, avoiding the use of multiple photodetectors and LED's. Lee et al. (2007) presented a waveguide block that avoided the use of fibers, though still used individual photodetectors and LED light sources for each sensor.

## Characterization and Modeling of DO Transfer

Traditionally, three methods have been widely used to measure rates of oxygen transfer from gas to liquid in bioreactors—direct measurement of composition and flow rate of inlet and exhaust gases with the difference in products giving the rate of transfer, consumption of DO by sulfites with measurement of sulfite concentration by sampling, and the dynamic or “gassing-out” method. The gassing-out method involves switching the head-space or sparging gas from air to nitrogen, and recording the DO profile as oxygen is transported out of the (initially air saturated) media. It is the only method feasible with sealed microbioreactor systems, and to date it is the only method that has been used in the literature to characterize microbioreactors, and nearly all minibioreactors—Hortsch et al.'s (2010) use of a modified sulfite method, with their unsealed system, being the exception.

The volumetric mass transfer coefficient is derived from the standard mass balance generated 1st order ODE model, with the assumption of a high degree of mixing and dominance of liquid phase mass transfer resistance.

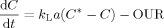
(1)
where *C* is the reactor DO concentration, and C* is the liquid phase concentration of oxygen at equilibrium with the gas phase concentration. The characteristic response time of this model during the gassing-out procedure, *τ*_m_, is given by 1/*k*_L_*a*. As the response time of DO sensors, *τ*_s_, is generally not ≪*τ*_m_, solutions of Equation (1) must be adjusted to compensate. Sensor behavior can be approximated as a 1st order system, giving the following solution for the gassing-out procedure, and allowing calculation of *k*_L_*a* from the sensor DO profile (*C*_s_).

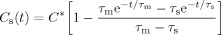
(2)

This assumes that the DO sensor measurement is representative of the spatial distribution of DO in the reactor however, that is, that there is a thin film where the gradient (mass transfer boundary layer) exists, and a well-mixed bulk with near uniform DO concentration. In some microbioreactor systems, this assumption may be questionable; certainly so for those that rely on diffusion for mixing. Almost all publications in this field, and the authors of this article, however have fitted this model to gassing-out DO profiles when calculating *k*_L_*a* values. This invites the question as to whether the *k*_L_*a* values generated from this curve fitting process are relevant in predicting DO transfer rates during bioreactor operation, that is, do they predict the same rate of DO transfer as you would expect during normal operation of the bioreactor?

Edge cases for mixing and cell distribution can give insight into the validity of the gassing-out approximation. Lee et al. (2006) examined the case of a planar, diffusion only microbioreactor with homogenous OUR and distribution of cells (see the Supplementary Information Section and Fig. S1). The maximum possible OUR for this system can be calculated at steady state by solution of the reaction–diffusion equation, and a corresponding *k*_L_*a* value can be derived. Then, if a single eigenmode dominates the solution for gassing-out procedure conditions, the reaction–diffusion equation can be reduced to the form of Equation (1). By retaining the dominant eigenvalue, the maximum OUR and corresponding *k*_L_*a* values for the dynamic gassing-out procedure are found. Comparison of the two *k*_L_*a* values shows a fairly close correspondence. The details of the procedures for this and for other edge cases are given in the Supplementary Information Section.

This method confirms the validity of *k*_L_*a* comparisons across scales, as the model effectively reduces to a form similar to Equation (1), and the predicted OUR and *k*_L_*a* values are similar. If cells settle out and there is no mixing however, a correction of ∼0.4 may need to be applied to the gassing-out *k*_L_*a*. Accordingly, caution should be exercised both when attempting scale up, and designing devices for cell types that are more prone to sedimentation—particularly if stagnant zones may be created by cellular fouling. Table II gives the gassing-out and steady state equivalent *k*_L_*a* and maximum OUR values for the four relevant cases.

**Table II:** Maximum OUR and *k*_L_*a* values predicted by the single eigenmode solution of the reaction–diffusion equation model for the dynamic gassing-out procedure 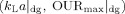, and values predicted by solution of the model for steady state fermentation conditions 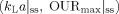.

	Steady state solution		Dynamic gassing-out solution	
Edge case	OUR_max_|_ss_	*k*_L_*a*|_ss_	OUR_max_|_dg_	*k*_L_*a*|_dg_
Perfect mixing	*k*_L_*a*|_dg_C^*^	*k*_L_*a*|_dg_	*k*_L_*a*|_dg_C^*^	*k*_L_*a*|_dg_ (measured)
Diffusion only; homogenous OUR; homogenous cell distribution	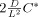	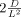	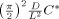	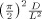
Diffusion only; point source OUR (at bioreactor bottom); all cells on bioreactor bottom	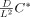	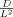	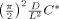	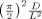
Well mixed; point source OUR (at bioreactor bottom); all cells on bioreactor bottom	*k*_L_*a*|_dg_C^*^	*k*_L_*a*|_dg_	*k*_L_*a*|_dg_C^*^	*k*_L_*a*|_dg_ (measured)

*L* refers to the characteristic length for diffusion in the liquid phase (typically the height of media in the bioreactor chamber), *D* to the diffusivity of oxygen in the medium, and C* to the liquid phase concentration of oxygen at equilibrium with the gas phase concentration. Well-mixed systems satisfy the assumptions of the 1st order ODE model presented as Equation (1), and thus all values can be calculated directly from the *k*_L_*a* value measured during the gassing-out procedure. Establishing the similarity between the two *k*_L_*a* values for the diffusion bioreactor with homogenous cell distribution/OUR is important, as it confirms the validity of using *k*_L_*a* measured in these devices for predicting their OTR performance, and validity of their use when comparing OTRs across volume scales. The scalar factor between the two values is ∼1.23. With the 3rd case, the diffusion bioreactor with heterogeneous cell distribution/OUR, steady state OTR performance is predicted to be less than half (∼0.41) of that expected from the measured *k*_L_*a* value. This strikes a note of caution, both when attempting scale up, and designing devices for cell types that are more prone to sedimentation—particularly if stagnant zones are created by cellular fouling. Details of this analysis are given in the Supplementary Information Section.

Matching oxygen transfer characteristics across scales is an important goal in process scale up. Accordingly, the ability to “tune” the *k*_L_*a* of a miniature bioreactor is desirable. As this process may involve many gassing-out experiments, correlations between *k*_L_*a* and tunable reactor/process variables could be quite advantageous. To date, such correlations have only been produced for shaken microwell plates. Doig et al. (2005) and Islam et al. (2008) have both produced engineering correlations using the canonical dimensionless groups to relate liquid mass transfer coefficient, *k*_L_*a*, and gas transfer area, *a*, to shaking frequency. It is foreseeable that similar correlations could be applied to stirred mini and microbioreactors, but the development of correlations for pneumatically and fluid driven systems is less clear. In these systems, fluid motion is generally produced by deformation of a membrane, which may be combined with injection/withdrawal of a fluid jet. There are no clear indicators of fluid velocity, so it is difficult to generate Reynolds numbers. Indeed, it may be that microparticle image velocimetry is required to correlate parameters such as oscillation frequency to Reynolds numbers, which in of itself would be a considerable experimental undertaking.

## OUR and DO Control

OUR and specific OUR (sOUR) have been used to characterize fermentation productivity and cellular metabolism for a variety of cell types (Santos et al., 2006; Zou et al., 2009), and have been considered as indicators of cell state/function (Deshpande and Heinzle, 2004; Ducommun et al., 2000; Kyung et al., 1994; Ozturk and Palsson, 1991) and viability (Deshpande and Heinzle, 2004; Konstantinov et al., 1994). Recently OUR has also been suggested as a screening tool for identifying more productive or promising strains (Alderman et al., 2004; Deshpande and Heinzle, 2004; Dumsday et al., 2009).

OUR can be calculated in real time from Equation (1), provided DO is monitored in real time and *k*_L_*a* values are stable over time. During fermentations the temporal gradient term can be generally be neglected, giving the following relationship.

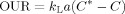
(3)
OUR may then be normalized by cell density, *X*, giving sOUR.

**Figure 4:**
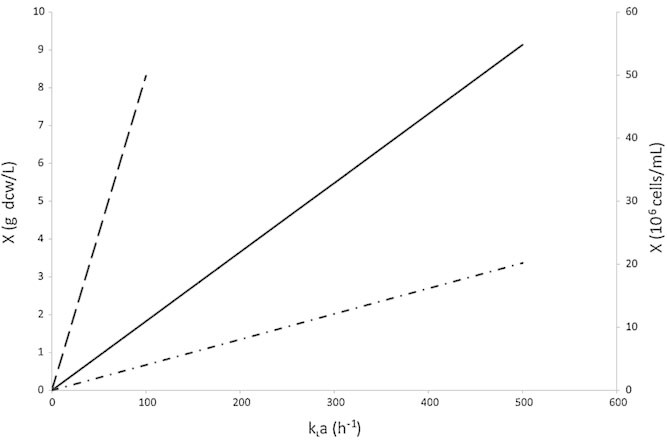
Maximum supportable cell densities for a 40% air DO set point. Solid line: *E. coli*, 20 mmol/g dcw/h was used for the sOUR value when calculating the maximum cell densities, as it has been reported as a typical sOUR for *E. coli* without oxygen limitation (Andersen and von Meyenburg, 1980). Dash-dot-dashed line: *S. cerevisiae*, 8 mmol/g dcw/h was used for the sOUR value when calculating the maximum cell densities, as it has been reported as a typical sOUR for *S. cerevisiae* without oxygen limitation (Sonnleitner and Käppeli, 1986). Dashed line: CHO cells, using the RHS vertical axis for cell density. 4.5 × 10^-15^ mol/cell/min was used for the sOUR value when calculating the maximum cell densities, as it has been reported as a typical sOUR for CHO cells without oxygen limitation (Nienow, 2006).

Examination of Equation (3) yields an interesting criterion. If control of DO is to be achieved, then the reactor must be able to transfer more oxygen than is consumed at the desired set point, *C*_sp_, that is, 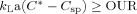. From this criterion and knowledge of typical sOUR values exhibited during the exponential phase, maximum cell densities for different set points can be calculated. Forty percent air is a typical DO set point in fermentation of many cell types. Figure 4 summarizes the maximum supportable cell densities for a range of *k*_L_*a* values.

## Characterization of Mixing

Fluid mixing is an important feature of any bioreactor. Micheletti and Lye (2006) have indicated in their review article that inhomogeneities, particularly with fluid addition, are the major source of variance between experiments. Mixing phenomena in microscale systems can be very complicated (Hessel et al., 2005), and has proved difficult to quantify—even with advanced dynamical treatments a definitive measure of degree of mixing has not resulted (Krasnopolskaya et al., 1999). The most useful and valid measures generally involve consideration of a measure of spatial variance in concentration of a dye (Krasnopolskaya et al., 1999).

These quantitative methods however are rarely implemented in experimental studies, and the requisite imaging equipment for considering reactor chambers in all three dimensions can be prohibitively expensive. Instead, a contrast dye, such as water blue, or a pH indicator, such as bromothymol blue, is often injected into the reactor chamber, and a time sequence of images are taken from below or above. Generally a time for “complete mixing” is estimated by eye. Unfortunately these methods do little to illuminate mixing behavior in the vertical direction, which is responsible for enhancing DO transfer and preventing cells from settling. As the *k*_L_*a* can be interpreted as a measure of the mass transfer boundary layer thickness in this direction, its enhancement over diffusion may offer a better indication of vertical mixing when more sophisticated treatments are not available.

Weiss et al. (2002) studied mixing phenomena in shaken 96-well plate wells, with 200 µL fluid volume, by injection of sodium hydroxide solution into wells either filled with a pH indicator, or with an immobilized pH sensitive layer at the bottom. Two approaches were used to study mixing: recording with a video camera from the side during, and monitoring of pH via a fluorescence plate reader after periods of shaking. Shaking conditions were also characterized using Reynolds and Froude numbers, and Büchs' phase number (Büchs et al., 2001). With the visual observations mixing times varied between 4 s with low volume injection and 900 rpm shaking frequency, and 3 min with higher volume injection and no shaking. Mixing time generally decreased with increasing shaking frequency, and thus increasing Reynolds and Froude numbers. Zones of high turbulence and rapid mixing were confined to the upper part of the well. Static conditions were observed towards the base, with dead zones noticed near the center of the well. The concept of zones of mixing was extended to a model that partitioned the fluid volume into 21 zones, with fluid exchange between them. Good correspondence with the pH monitoring results was reported, accounting for mixing times which were typically on the order of several minutes. These results may prove useful for designers and operators of bioreactors with similar geometry and volume to 96-well plates.

It should be noted that computational fluid dynamics (CFD) studies offer an alternative method of characterizing and enhancing mixing in bioreactor designs, but little work is available comparing simulated with empirical performance.

## Analysis of Published Miniaturized Bioreactor Systems

Two natural classes of miniaturized bioreactors have evolved—those with submilliliter volume, and those larger. The former have been between 5 and 800 µL in volume and, further differentiating them from the latter, have been sealed and aerated via gas permeable poly(dimethylsiloxane) (PDMS) membranes. The larger systems, or minibioreactors, are typically between 1 and 10 mL in volume, and are agitated by stirring.

### Microbioreactors

**Figure 5:**
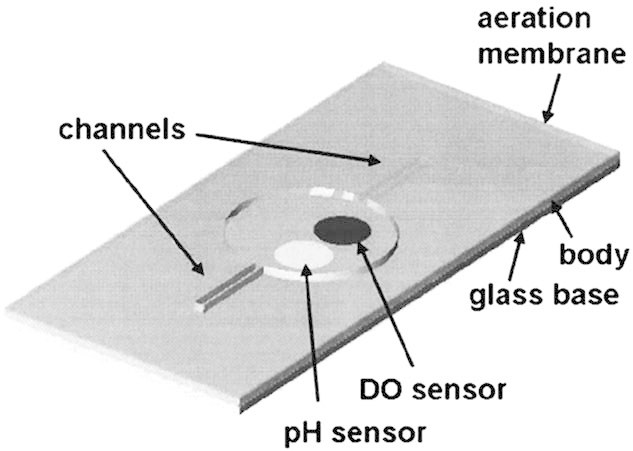
The diffusion dependent 50 µL microbioreactor developed by Zanzotto et al. (2004) as the first microbioreactor to support cell culture. The system is comprised of three PDMS layers on a glass base. The reactor chamber is 300 µm deep and bounded by a 100 µm thick PDMS aeration membrane. Fluorescence lifetime sensor spots are visible; OD is measured vertically through the chamber. Figure reproduced with permission from Zanzotto et al. (2004) (Copyright 2004 Wiley & Sons Ltd.).

Zanzotto et al. (2004) introduced the 1st of these microbioreactors, with 5 and 50 µL volume PDMS devices that relied on diffusion for mixing and mass transfer, and were aerated through a 100 µm thick PDMS membrane. The devices used commercially available fluorescence lifetime sensor spots to measure DO and pH, while transmitted 600 nm light intensity was measured vertically through the reactor chamber to calculate optical density (OD), and hence cell density. Chamber height, the characteristic dimension for oxygen transfer, was 300 µm. A *k*_L_*a* of 60 h^-1^ was measured via the gassing-out method. This is considerably lower than the two limiting cases for diffusion systems predicted in Table II, 250 and 100 h^-1^, respectively, and also considerably lower than the dynamic simulation presented in Zanzotto et al. (170 h^-1^). The discrepancy was attributed to significant oxygen transfer resistance from the PDMS membrane. Other factors may be responsible, such as a bulging membrane effectively increasing the chamber height. In 10 h duration batch fermentations with *E. coli* the system supported ODs of ∼5 (∼4 × 10^9^ cells/mL) with air, and ∼6 with pure oxygen flushing of the head space. DO depletion with air was typically seen after 4 h at an OD of ∼1. The device, illustrated in Figure 5, was used in two further *E. coli* studies, with integrated bioluminescence and fluorescence monitoring (Zanzotto et al., 2006), and endpoint gene expression analysis (Boccazzi et al., 2005).

Active mixing of microbioreactors soon followed. Zhang et al. (2006b) developed a 150 µL volume magnetic stir bar mixed system and claimed to induce behavior corresponding to the transitional regime between laminar and turbulent flow. The reactor, fabricated from a sandwich of PDMS and poly(methylmethacrylate) (PMMA) parts, featured a 10 mm diameter × 1 mm thick PDMS chamber bounded by a 100 µm PDMS aeration membrane which was allowed to bulge out to ∼2 mm (total reactor chamber depth). A 6 mm long stir bar was positioned in the chamber and spun at speeds from 180 to 850 rpm. At 850 rpm the rotational Reynolds number, *Re*_*n*_, was 130, exceeding the transition number of 100 observed for STRs by Biń (1984) Complete mixing within 30 s at 180 rpm was reported, as were *k*_L_*a* values from 20 to 75 h^-1^ over the stirring speed range of 200–800 rpm. *k*_L_*a* values exhibited a steady increase over this range, but were not correlated to the Reynolds number or other dimensionless groups. Microfluidic channels connected the reactor chamber to the “macroscopic” world for inoculation and other functions. Instrumentation was similar to that used by Zanzotto et al. (2004).

**Figure 6:**
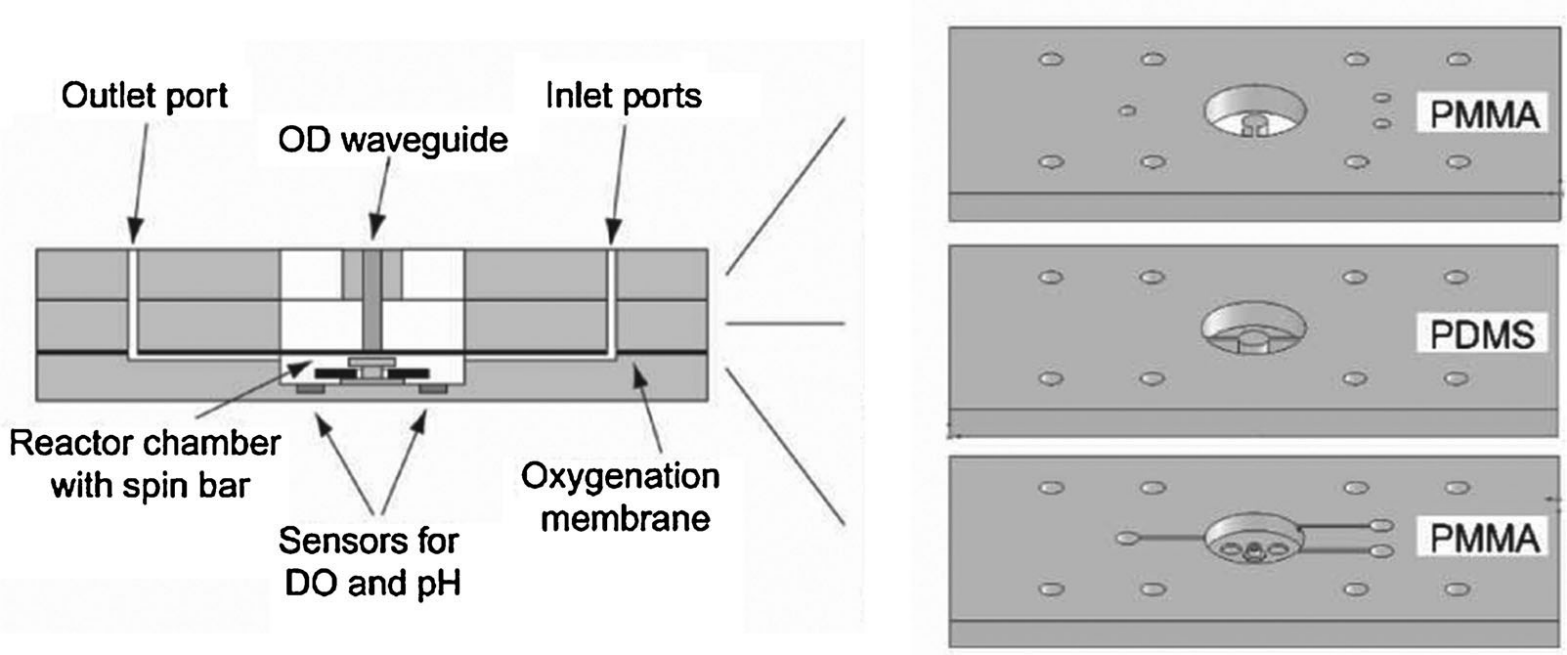
Schematic representation of magnetic stir bar mixed 150 µL microbioreactor presented by Szita et al. (2005). The system is comprised of two layers of PMMA and one layer of PDMS. PMMA and PDMS waveguides are visible for OD measurement. Fluidic ports were connected to the reactor chamber by microfluidic channels. Figure reproduced with permission from Szita et al. (2005) (Copyright 2005 Royal Society of Chemistry).

Batch fermentations were undertaken with *E. coli*, resulting in final OD's of ∼6 after 16 h, and oxygen depletion times of ∼2.5 h at OD ∼2, with 700 rpm agitation. Impressively, this data corresponded well with results obtained in 500 mL bench-scale SixFors® reactors (Infors AG, Switzerland), as well as those from shaken flasks and test tubes. Fermentations were also completed with *Saccharomyces cerevisiae*, resulting in OD's of ∼6 after 30 h, and oxygen depletion after 10 h at OD ∼2. Stirring speed was again 700 rpm. The system, illustrated in Figure 6, was modified slightly by increasing chamber height to 2 mm and integrating a grid above the membrane to prevent bulging. In this modified form it was used in two further studies—differential end point gene expression studies for *S. cerevisiae* grown in galactose and glucose media (Boccazzi et al., 2006), and more significantly, the 1st presentation of a multiplexed array of microbioreactors (Szita et al., 2005). The four microbioreactors were mounted on a platform, and the instrumentation was multiplexed by optical fibers on a bracket connected to a mechanical slider, driven by an electromagnetic motor. The bracket moves along the slider from one reactor to the next in series, reducing the number of optical components required, but reducing the frequency with which parameters can be measured (sampling period ∼10 min). In neither study was the modified reactor characterized for *k*_L_*a*.

Stirred mixing leads to another application—approximation of a CSTR. Indeed, this application may be the main advantage of magnetically stirred microbioreactors. Though industrial bioprocesses are typically carried out in batch or fed-batch mode, chemostat operation is desired by many for the quality of kinetic data it can produce (Nielsen et al., 2003). Other mixing techniques have achieved greater OTRs and seemingly faster mixing (Lee et al., 2006), but CSTR operation may require greater complexity, due to the influence of the mixing technique on any attempted continuous fluid flow. Another 150 µL device, very similar to the 2 mm thick chamber devices described above, was developed as a chemostat (Zhang et al., 2006a). In *E. coli* fermentations the reactor was successfully operated for 180 h at an OD of 1, at dilution rates up to 1.5 h^-1^. Neither stirring speed nor *k*_L_*a* were reported. Additionally, the devices fluid contacting surfaces were grafted with an anti-fouling co-polymer comb layer. Though this is an interesting and impressive development, it would be a potentially costly and difficult manufacturing step if necessary for satisfactory device operation.

**Figure 7:**
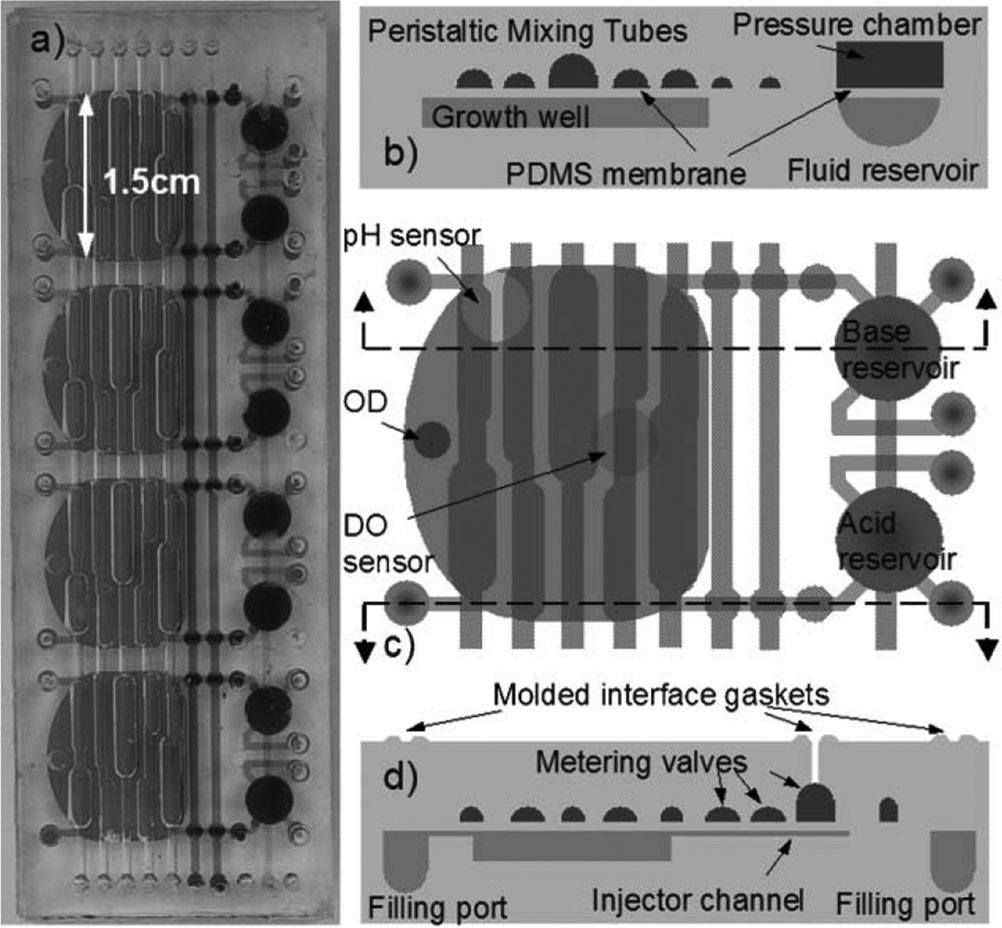
Pneumatic peristaltic 100 µL microbioreactor system presented by Lee et al. (2006). a: Four reactor module image showing common pneumatic channels. b: Bioreactor cross-section illustrating pressure actuated channels for mixing, and pressurized acid/base reservoir. c: Top view of single reactor with sensors indicated, and peristalsis layout. d: Cross-section showing metering valves for releasing acid/base solution. Figure reproduced with permission from Lee et al. (2006) (Copyright 2006 Royal Society of Chemistry).

A significant development came with the introduction of an array of eight peristaltically mixed 100 µL PDMS microbioreactors that achieved a high *k*_L_*a* of 360 h^-1^ (Lee et al., 2006). The reactor chambers were 500 µm high, an irregular oval shape ∼15 mm in maximum dimension, and bounded by a 70 µm thick PDMS membrane. A series of channels above the membranes could be pressurized with 4 psi air or a gas mix, deflecting the membrane into the reactor chamber. When actuated at 40 Hz frequency in a propagating pattern, mixing times of ∼5 s were recorded, along with the 360 h^-1^ *k*_L_*a*. This system produces high DO transfer, and also has the advantage of no moving reactor parts—all actuation is driven by deformation of integrated PDMS membranes by off chip pressure, which is controlled by relatively simple original equipment manufacturer (OEM) microvalves. Custom DO and commercially available pH sensor spots were used, with a modified detection fiber for OD with a claimed linear range up to 40. The modification, based on the work of Hodkinson (1966) and Swanson et al. (1999), uses two pinholes to restrict the angle of entry to the detection fiber to less than 1/10th of the first angular minimum for Fraunhofer diffraction, reducing the likelihood of multiply scattered light being scattered back into it, and hence the detector. DO control was attempted by varying the composition of the 4 psi gas mix delivered to the peristaltic mixing channels, and pH was controlled by injecting acid or base from pneumatically actuated reservoirs. The system is illustrated in Figure 7.

Eight-hour batch cultures were undertaken with *E. coli*, reaching ODs of ∼40 (13.8 g dcw/L) with pH control and DO control. DO control parameters were set to maintain a concentration above that equivalent to 50% air saturation. After 3 h DO fell to ∼40% air and was maintained, but with considerable variance. pH control was more consistent, and in only pH controlled fermentations oxygen depletion was seen after 4 h at ∼6 g dcw/L biomass. This was the first published system offering control over variables other than temperature. DO control complications may have arisen from using a single valve to control the gas mix for four reactors, or the complication of using two algorithms together to specify gas mix and pressure cycling through peristalsis channels. It is notable however that the gas mix pulse width modulation (PWM) frequency was 0.1–3 Hz—possibly insufficient in the authors' experiences for controlling a gas mix.

A related system was developed as a single chemostat/turbidostat microbioreactor with 1 mL working volume (Lee et al., 2011). Fabricated from PC, with PDMS membranes for deformable components, it utilized three horizontally split, 2 mm thick, 500 µL chambers which were linked with channels and deformed pneumatically to induce peristaltic motion between them. The layout reduced the diffusion area available through the 65 µm aeration membranes, with *k*_L_*a* values of 57.6–90 h^-1^ reported. Mixing times of 2 s were claimed. All fluid handling was integrated on chip via pneumatic valves, reservoirs, and peristaltic pumps. Surfaces were modified as in Zhang et al. (2006a) 3 weeks of continuous fermentation with *E. coli* was maintained, with DO and pH control achieved. OD values were typically 1–2 (0.3–0.7 g dcw/L), but reached as high as 4 (1.3 g dcw/L). Dilution rate was typically ∼0.3 h^-1^. Turbidostat control variance was reported as <1.2%. Gas mixes for DO control were varied at 10 Hz. Comparison of DO control variance with the earlier eight microbioreactor array is difficult due to the different time scales.

Funke et al. (2009) combined an optical monitoring system (“BioLector”) for DO, pH, and back scattered light (as a cell density indicator) with a 48-well format microtiter plate that employed a variety of baffles to maximize OTR. This system allowed *k*_L_*a* values >600 h^-1^ for a 500 µL culture volume, and sequential monitoring of individual wells via an automated *x*–*y* stage. The system was further developed as a unit with four un-baffled round wells, via the addition of a microfluidic base layer which allowed pneumatic dosing from two reservoirs, for either pH controlled or fed-batch operation (Buchenauer et al., 2009). This system lacked DO monitoring however. A new system variant (“Microfluidic BioLector”) with DO monitoring and an improved microfluidic pumping mechanism was described in 2010 (Funke et al., 2010b). For *E. coli* culture, its four 12 mm diameter wells were inoculated with 500 µL of media, with culture performed under uncontrolled, pH controlled, and glucose fed-batch conditions. The tape-sealed wells have a capacity of 2 mL, though this is limited by fluid wall climbing and splashing at higher filling volumes (Funke et al., 2009). A shaking speed of 800 rpm was used, with 3 mm shaking diameter. Higher shaking speeds, and hence OTRs, were available, but shaking may have been limited by the fluid wall climbing effect's tendency to reduce fluid height in the center of wells. This may also reduce the path length for light scattering measurements below a critical value (Funke et al., 2009). Though *k*_L_*a* was not measured for this study, the operating conditions correspond to a value of ∼170 h^-1^, based on previous measurements of a similar system (Funke et al., 2009). In batch operation exponential growth was observed, with DO depletion after ∼3.5 h. Neither biomass concentration nor OD can be reported at this point as relative back scattering density was monitored as the indicator of cell density. Higher end point relative back scattering density was observed with pH control. Fed-batch cultures began in minimal media without glucose, which was added at a constant rate. Accordingly, DO depletion did not begin until later, with higher end point relative back scattering density than in the batch cultures recorded. End point cell densities were not reported. A final study was performed in the Microfluidic BioLector system, with shaking speed at 1,000 rpm, delivering a *k*_L_*a* of 460 h^-1^ in baffled wells (Funke et al., 2010a). *E. coli* culture produced similar results to bench-scale fermentations when *k*_L_*a* values were matched across scale.

Published microbioreactor studies are summarized in Table III.

**Table III:** Summary of microbioreactor properties reported in peer reviewed publications.

Refs.	*k*_L_*a* (h^-1^)	Volume (µL)	DO transfer dimension (µm)	Mixing	Cell type	Measured	Controlled	Notes
Zanzotto et al. (2004)	60	5, 50	300	Diffusion	*E. coli*	DO, OD, pH	T	
Boccazzi et al. (2005)		50	300	Diffusion	*E. coli*	DO, OD, pH	T	
Szita et al. (2005)		150	2,000	Magnetic stir bar	*E. coli*	DO, OD, pH	T	Multiplexed
Zhang et al. (2006b)	20–75	150	∼2,000	Magnetic stir bar	*E. coli*, *S. cerevisiae*	DO, OD, pH	T	
Zhang et al. (2006a)		150	2,000	Magnetic stir bar	*E. coli*	DO, OD, pH	T	Chemostat
Boccazzi et al. (2006)		150	2,000	Magnetic stir bar	*S. cerevisiae*	DO, OD, pH	T	
Zanzotto et al. (2006)		50	300	Diffusion	*E. coli*	DO, OD, pH	T	
Lee et al. (2006)	360	100	500	pneumatic Peristaltic	*E. coli*	DO, OD, pH	T, DO, pH	Multiplexed
Funke et al. (2010b)	170	500	4,400	Shaken device	*E. coli*	DO, pH, backscattered light	T, pH	Fed batch, multiplexed
Funke et al. (2010a)	460	500	4,400	Shaken device	*E. coli*	DO, pH, backscattered light	T, pH	Fed batch, multiplexed
Lee et al. (2011)	58–90	1,000	1,000	Pneumatic peristaltic	*E. coli*	DO, pH, OD	T, DO, pH, OD	Chemostat, turbidostat

### Minibioreactors

Kostov et al. (2001) integrated custom DO and pH sensor spots, an OD measurement, and a magnetic stir bar in a 4 mL polystyrene (PS) cuvette, initiating research into miniaturized bioreactors. Their system had a working volume of 2 mL, was aerated by gas flow from the cuvette top, and stirred at 300 rpm. This gave it a *k*_L_*a* of 21 h^-1^. In 25 h *E. coli* fermentations OD's of ∼6 were reached, with DO depletion after ∼4 h at ∼OD 3. Unlike the microbioreactors above, the DO sensor was positioned at the vertical midpoint of the working volume, which may have led to higher values of DO being recorded.

Most subsequent developments were in larger systems such as Lamping et al.'s 6 mL working volume Rushton turbine, sparged bioreactor (Lamping et al., 2003). This combined a miniaturized version of large scale bioreactor agitation equipment with commercial fiber optic probe DO and pH sensors, and fibers for 625 nm OD measurement in a PMMA cylindrical system 48 mm high × 16 mm in diameter. *k*_L_*a* values from 70 to 360 h^-1^ were achieved, but *E. coli* fermentations were carried out at 100 h^-1^. Biomass concentrations of 1.6 g dcw/L were achieved after 10 h, with DO depletion after 2 h at ∼0.7 g dcw/L. Both results were similar to those obtained in a 15 L fermenter.

Harms et al. (2006) followed with two 24 reactor, 1 mL working volume, stirred-tank systems based on the geometry of 24-well plate wells. One system was based on an unsealed 24-well plate layout and used a single electronics package to interrogate reactors one at a time. The other was comprised of discrete reactors in a rotary configuration, with each having dedicated monitoring circuitry, enabling simultaneous interrogation of reactors. Each discrete reactor was sealable by a removable cap. The sensor packages included optical monitoring of DO, OD, pH, and GFP expression levels. *k*_L_*a* values >100 h^-1^ (ranging from ∼68 to 300 h^-1^) were obtained, but large variances in these were observed, particularly with impeller speeds of 1,000 rpm or higher. Both systems were sparged, but the discrete reactor system was also run with just surface aeration, for which similar results to sparging were claimed. *E. coli* cultures were performed, with oxygen depletion seen after 2 h. Neither biomass concentration nor OD can be reported at this point due to problems with OD monitoring, which suffered from low signal to noise ratios, perhaps due to complications with bubble formation. Some DO control was attempted via on/off switching of agitation speeds from high to low. As with all sparged small volume systems, foaming may be a concern.

The individual reactors were further developed as a 12.5 mL system, upon which mixing studies were performed (Vallejos et al., 2006). Subsequent work focused on systems of volume >30 mL (Ge et al., 2006; Hanson et al., 2009; Kondragunta et al., 2010).

**Figure 8:**
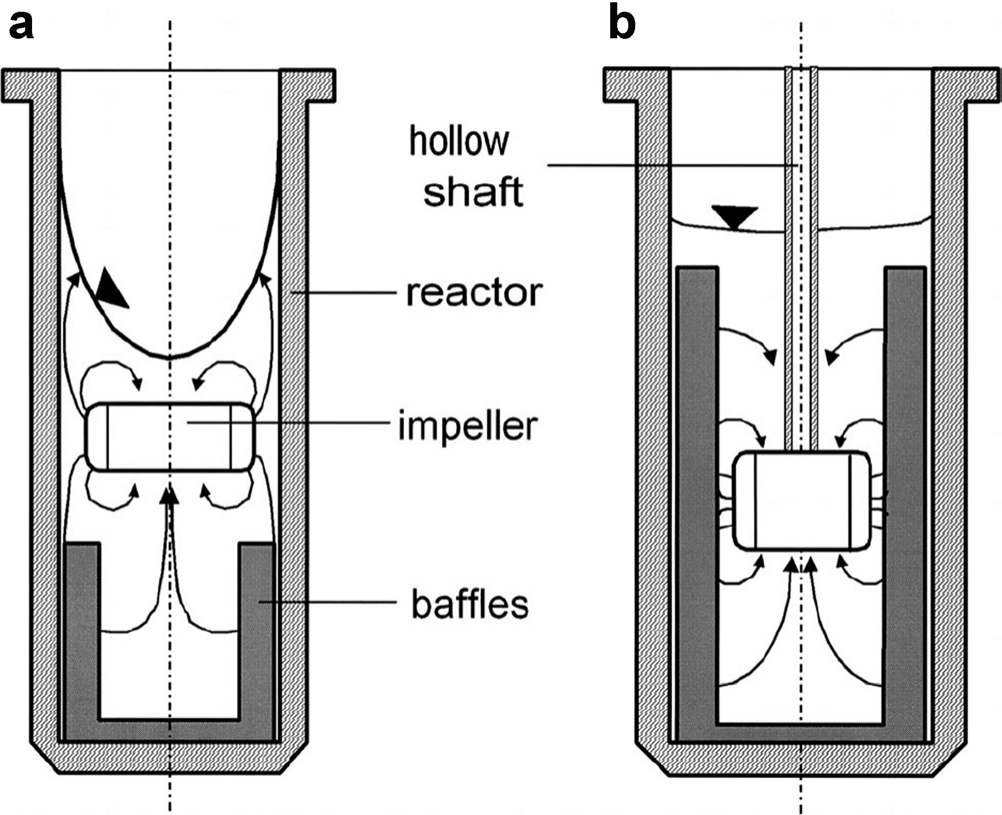
Schematic illustrations of impeller induced mixing in minibioreactor chambers in an array presented by Puskeiler et al. (2005). Design b included a hollow shaft for sparging and produced superior DO transfer. *k*_L_*a* values reported in this review were produced using design b. Figure reproduced with permission from Puskeiler et al. (2005) (Copyright 2005 John Wiley & Sons Ltd.).

Puskeiler et al. (2005) developed a system of 48 sparged and agitated, 20 mm diameter × 86 mm high, baffled PS reactors, that typically operated between 5 and 12 mL volumes. The sparging and baffles design allowed for very high *k*_L_*a* values to be obtained, from 180 to 1,440 h^-1^ at the highest agitation speed (2,300 rpm). This arrangement is outlined in schematic form in Figure 8. The system was integrated with a robotic fluid handling system from Tecan (Männedorf, Switzerland), which allowed for sampling and addition of fluids, enabling pH control and measurement, periodic OD measurements, and fed batch operation. In a subsequent publication (Weuster-Botz et al., 2005), integration with a Presens (Regensburg, Germany) DO sensor block was described, which allowed online DO measurement. Batch fermentations of *E. coli* operated at the highest agitation speeds produced maximum cell densities of 16.5 g dcw/L after 7 h, with DO depleted to 25% air after ∼4.5 h, at ∼13.5 g dcw/L biomass (Weuster-Botz et al., 2005). Notably, this was a similar density to that obtained by Lee et al. (2006) with their 100 µL scale system. Fed batch operation produced densities up to 20.5 g dcw/L. The reactor block design has drawbacks though—integration of fibers or other photonic elements, required for measurements such as OD, is difficult and, as with other open or sparged systems, dehydration and foaming may be problems. Additionally, relying on sampling for cell density and other measurements limits measurement frequency considerably.

This system was further extended to support culture of the filamentous actinobacteria *Streptomyces tendae* (Hortsch et al., 2010). This involved the use of a one-sided impeller which provided better gas–liquid mass transfer in the more viscous culture medium, and generated a rotating lamella which minimized wall growth. *k*_L_*a* was estimated at ∼180 h^-1^ for the operating conditions. The wells were surface aerated rather than sparged, reducing bubble formation and avoiding foaming. Bench-scale comparable results were obtained for cell growth, carbon source consumption, and antibiotic production over ∼200 h culture time, with maximum biomass reaching ∼20 g dcw/L. DO did not fall below ∼50% air saturation, reaching this level at ∼12 g dcw/L biomass. DO profiles and *k*_L_*a* values were not disclosed for the bench-scale system. Stirrer power dissipation was considered at both scales, with more uniform viscous energy dissipation in the minibioreactors—an advantage when culturing shear sensitive cell types.

In 2006 Microreactor Technologies, Inc. (Mountain View, CA) introduced the Micro-24 system. Supporting up to 10 mL per reactor, it is fabricated in a 24-well plate format polymer system, has integrated fluorescence lifetime DO and pH sensors, is sparged with a gas mix or acidic/basic gases through a bottom membrane for DO and pH control, is sealed by gas-permeable adhesive tape, and is mounted on a shaken sensor block for agitation and sensor interrogation. Monitoring of cell density requires manual sampling from reactors. The system was first characterized by Tang et al. (2006) for culture of the bacterium *Shewanella oneidensis*, but DO transfer was not characterized. DO control at 15% air saturation set point was reported, as was pH control. An OD of ∼1 was reached after 20 h. A second DO control scheme that maintained concentration above 20% air, via an oxygen enriched feed gas, was used, with a slightly higher OD of ∼1.3 reached.

Isett et al. (2007) conducted 5 mL working volume fermentations in the Micro-24 system for *E. coli*, *S. cerevisiae*, and *Pichia pastoris*. *k*_L_*a* values of 32.6–56.1 h^-1^ were reported over the 500–800 rpm shaking speed range. Higher *k*_L_*a* values were not possible due to the tendency of sparging to cause excessive foaming. Mixing times from >100 to ∼1 s were claimed. *S. cerevisiae* was cultured in 40 g/L dextrose media to ∼OD 14 (8 g dcw/L) after 35 h. DO bottomed after 25 h at ∼10% air and OD 12 (6 g dcw/L). The low biomass yield on dextrose and relatively high cell density at DO depletion, given the reported *k*_L_*a* values, may have been due to anaerobic glucose metabolism in the high carbon source media. DO and pH control were attempted with *E. coli*, via infusion of oxygen and ammonia gasses respectively. OD reached ∼23, and DO declined to the below the intended 30% air lower limit to ∼15% air after 3 h, at OD ∼3. pH control did not appear to be successful. DO and pH control were reported with *P. pastoris*, but the 20% air set point was accompanied by significant variance and noise. Cell density data were difficult to interpret.

Finally, Chen et al. (2009) reported Chinese hamster ovary (CHO) cell culture in the device. DO transfer was not characterized, and DO control was not used due to sparging induced foaming. A maximum cell density of ∼7 × 10^6^ cells/mL was reported, with no DO depletion due to the low OUR requirements of CHO cell fermentations. This was the first published study of CHO cell cultivation in a miniaturized bioreactor. In 2004 though, Deshpande et al. (2004) monitored OUR during 200 µL CHO cell cultures in a shaken 96-well microplate with integrated fluorescence lifetime DO sensors. Cell density was not monitored or published, and *k*_L_*a* was not measured.

Klein et al. (2012) characterized the most recent system described in the literature—an array of eight parallel 10 mL working volume bioreactors, based on 16 mm internal diameter Hungate tubes. Developed from an earlier described (Nanchen et al., 2006), perfused, sparged tube system that lacked integrated monitoring, these chemostat bioreactors were sparged with air and stirred via magnetic corrugated disc stirrers. The tubes were sealed with a septum perforated by three tubes for sparging, effluent, and liquid feed. Biomass and other concentrations were determined from the liquid effluent, with off-gasses used to monitor CO_2_ production and OUR. DO concentration was monitored via fluorescence lifetime sensor spots on the vessel vertical walls. *k*_L_*a* was reported as 26.8 h^-1^ at 1,000 rpm stirring rate and 48.5 h^-1^ at 2,000 rpm. A chemostat mode fermentation with a *Schizosaccharomyces pombe* yeast strain was performed over a period of 80 h with a dilution rate of 0.1 h^-1^. DO never fell below 60% air during it, with biomass concentration typically ∼1.6 g dcw/L.

Published minibioreactor studies are summarized in Table IV.

**Table IV:** Summary of minibioreactor properties reported in peer reviewed publications.

Refs.	*k*_L_*a* (h^-1^)	Volume (mL)	Filling height (mm)	Mixing	Cell type	Measured	Controlled	Notes
Kostov et al. (2001)	9.8–21	2	20	Magnetic stir bar + sparging	*E. coli*	DO, OD, pH	T	Foaming problems
Lamping et al. (2003)	70–360, 100 used	6	30	Impeller + sparging	*E. coli*	DO, OD, pH	T	Difficult to multiplex
Puskeiler et al. (2005)	180–1,440	5–12	32 (at 10 mL)	Impeller + sparging	*E. coli*		T, pH	No real-time OD, DO, automated sampling for OD
Weuster-Botz et al. (2005)		12	38	Impeller + sparging	*E. coli*	DO, OD, pH	T	Multiplexed DO monitoring added, automated sampling for OD
Harms et al. (2006)	68–300	1	5.2	Impeller + sparging	*E. coli*	DO, OD, pH, GFP		Problems measuring OD, GFP, *k*_L_*a* inconsistency at higher speeds
Tang et al. (2006)		6	31.4	Agitated	*Shewanella oneidensis*	DO, pH	T, DO, pH	Manual sampling for OD, foaming problems
Isett et al. (2007)	32.6–56.1	5	26.2	Agitated	*S. cerevisiae*, *E. coli*, *P. pastoris*	DO, pH	T, DO, pH	Manual sampling for OD, foaming problems
Chen et al. (2009)		6	31.4	Agitated	CHO	DO, pH	T	Manual sampling for OD, foaming problems
Hortsch et al. (2010)	180	10	3.2	Impeller	*Streptomyces tendae*	DO, pH	T	Manual sampling for OD, product concentration
Klein et al. (2012)	26.8–48.5	10	50	Magnetic stir disc	*Schizosaccharomyces pombe*	DO	T	Chemostat, off gas and liquid effluent analysis

## Conclusions

Micro- and minibioreactors have been demonstrated to support fermentation of prokaryote and eukaryote microbes over a relevant range of biomass densities, with oxygen transfer characteristics comparable with bench, pilot, and production scale systems.

Microbioreactors, Puskeiler et al. (2005) aside, have generally displayed higher *k*_L_*a* values, and have offered as much if not more integration with online instrumentation and control. Indeed, often the design of the larger systems inhibits integration of optical fibers for absorption or bio-luminescence/fluorescence measurements. As these systems have produced similar results to larger scale bioreactors, and their *k*_L_*a* values have been as high as 460 h^-1^, microbioreactors may be considered an adequate micro-scale analogue of mainstream process development bench-scale bioreactors—creating a niche where they offer long-term, information rich screening with similar function. Microbioreactors, have been largely limited to *E. coli* to date however. Conversely, mini-scale systems have supported many other cell types including yeasts, filamentous actinobacteria, and mammalian cells. Though *k*_L_*a* values of up to 1,440 h^-1^ have been demonstrated, minibioreactors typically report lower OTRs than microbioreactors.

Fluid handling also remains an issue with microbioreactors. There has been little in the way of sampling, or integration with automated fluid handling systems—a particular limitation when determining product titer over time. Mostly sealed systems, they are typically limited to batch and chemostat modes of operation, though fed-batch operation has been demonstrated by Buchenauer et al. (2009). Minibioreactors generally offer fed-batch and batch modes, though Klein et al. (2012) have recently demonstrated a chemostat device. A final drawback for microbioreactors comes when considering reusability—they are suitable for multiplexing and are disposable, but the cost of fluorescence lifetime sensors needs to be taken into account if used in this manner. The eventual development and integration of glucose and other sensors will enhance their roles in process development though.

In analyzing models and characterization techniques for DO transfer, we have established that *k*_L_*a* is a suitable single value criterion for comparing performance from the microliter to production scales. Additionally, a correction factor has been derived for systems where cells have settled out in the reactor chamber. The possibility of real-time monitoring of OUR and its effect on DO control have been analyzed, and a deficit in quantifying mixing behavior in reactor chambers has been identified.

Finally, various DO sensor systems have been analyzed, with fluorescence lifetime systems remaining the only feasible option.
