# Supplementary figures and images for: Oxygen Transfer Characteristics of Miniaturized Bioreactor Systems
Source: Biotechnol Bioeng. 2013 Jan 17;110(4):1005–19. doi: 10.1002/bit.24824 (PMC3790518; doi:10.1002/bit.24824)

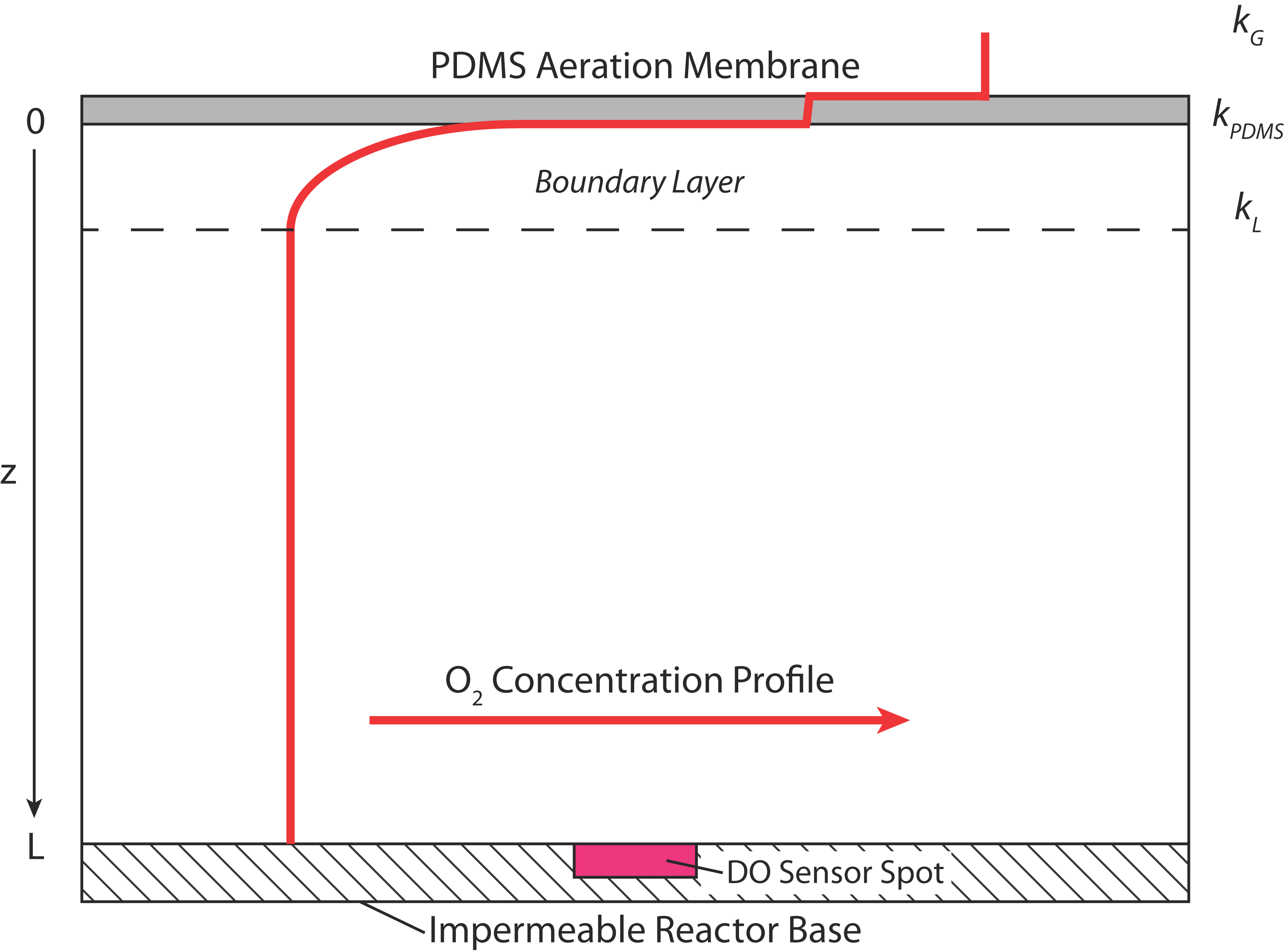

Supplement: Supplementary file 1 [file bit0110-1005-SD1.tif]
