# Supplementary material for: Oxygen Transfer Characteristics of Miniaturized Bioreactor Systems
Source: Biotechnol Bioeng. 2013 Jan 17;110(4):1005–19. doi: 10.1002/bit.24824 (PMC3790518; doi:10.1002/bit.24824)
Supplement: Supplementary file 2 [file bit0110-1005-SD2.doc]

**SUPPLEMENTARY MATERIAL**

The volumetric mass transfer coefficient, k_L_a, is the characteristic parameter of the standard mass balance generated 1^st^ order ODE.

$$\frac{dC}{dt}=k_{L}a\left(C^{*}-C\right)-OUR$$

This assumes sufficient mixing occurs, that gaseous species are at equilibrium across interfaces, fluid volume is constant, and that liquid phase mass transfer dominates. This scenario is illustrated in figure S1. $C$ refers to the concentration of oxygen in the liquid phase and $C^{*}$ to the liquid phase concentration of oxygen at equilibrium with the gas phase concentration. OUR refers to the oxygen uptake rate of the mass of cells in the bioreactor.

In some microbioreactor systems, this mixing assumption may be questionable; certainly so for those that rely on diffusion for mass transport. Almost all publications in this field, and the authors of this paper, however have fitted this model to gassing-out DO profiles when calculating k_L_a values. This invites the question as to whether k_L_a values generated from this curve fitting process are relevant in predicting dissolved oxygen transfer rates during bioreactor operation. i.e. do they predict the same rate of oxygen transfer as you would get at steady state during normal operation of the bioreactor?

To answer this question, we need to solve for the oxygen uptake rate (OUR) at steady state for each case of bioreactor operation, and compare these values to that predicted by the k_L_a value found from the dynamic gassing-out procedure. For convenience we compare the maximum supported OUR values (OUR_max_). i.e. OUR at steady state when the measured DO concentration is zero, which gives the highest rate of oxygen transfer.

Solving equation at steady state allows calculation of OUR as follows:

$$OUR=\left.k_{L}a\right|_{ss}\cdot\left(C^{*}-C\right)$$

OUR is maximised when $C=0$, giving the following relationship:

$$OUR_{\max}=\left.k_{L}a\right|_{ss}\cdot C^{*}$$

This relationship also shows how an “equivalent” k_L_a could be calculated, that corresponds to the OUR_max_ value for a given mode of bioreactor operation. i.e. the k_L_a needed in equation (1) to support that OUR_max_.

A schematic view of a typical aeration membrane microbioreactor is shown in figure S1. When diffusion dominates, mass transport in such a bioreactor can be described by the 1-D Cartesian coordinates reaction-diffusion model (equation ).

$$\frac{\partial C}{\partial t}=D\frac{\partial^{2}C}{\partial z^{2}}-OUR\left(C\right)$$

Solving equation for maximum OUR at steady state requires the following boundary conditions: $C(0,t)=C^{*}$ and $C(L,t)=0$. Cells are assumed to be equally distributed, and OUR is taken as a constant. This gives:

$$OUR_{\max}=2\frac{D}{L^{2}}C^{*}$$

$L$ refers to the characteristic length for diffusion in the liquid phase (here, the height of the bioreactor chamber), and $D$ to the diffusivity of oxygen in the medium. From equation this implies that the equivalent k_L_a at steady state, $\left.k_{L}a\right|_{ss}$, is given as follows:

$$\left.k_{L}a\right|_{ss}=2\frac{D}{L^{2}}$$

Now that OUR_max_ for a diffusion bioreactor has been predicted from the analytical steady state solution, we need to compare it to that predicted by the dynamic gassing-out procedure.

Lee et al. demonstrated that a diffusion equation model of the dynamic gassing-out procedure can be approximated by an equation with the form of equation , by making an eigenmode expansion and retaining only the 1^st^ eigenmode. The reduction from the reaction-diffusion model (equation ) is shown below.

In the separation of variables method for solution of the diffusion equation the eigenvalues, $\lambda$, are defined such that $\frac{\partial^{2}C}{\partial z^{2}}=-\lambda^{2}C$. Substituting into equation gives the following:

$$\frac{dC}{dt}=-D\lambda^{2}C-OUR$$

Equation (1), the mass balance derived governing ODE for a well-mixed bioreactor, considers the difference between $C$ and $C^{*}$ as the driving force. Substituting this difference in place of C in equation gives the familiar form.

$$\frac{dC}{dt}=D\lambda^{2}\left(C^{*}-C\right)-OUR$$

From equation it is clear that $D\lambda^{2}$ is the equivalent k_L_a in this approximation of the dynamic gassing-out procedure, $\left.k_{L}a\right|_{dg}$. This approximation is somewhat analogous to curve fitting (ie to approximating) equation to the dynamic gassing-out procedure DO profile. This gives the following relationship between predicted OUR_max_ and $\left.k_{L}a\right|_{dg}$ at steady state:

$$OUR_{\max}=\left.k_{L}a\right|_{dg}\cdot C^{*}=D\lambda^{2}\cdot C^{*}$$

For the relationship in equation to remain a valid approximation one eigenmode must dominate. If the dynamic gassing-out procedure is considered as a decay process, then the decay spectrum is given by these eigenmodes. These are found by applying a separation of variables and Fourier series solution procedure to the diffusion equation.

Consider the 1-D Cartesian microbioreactor model from equation again. For dynamic gassing-out characterisation the ambient gas phase is rapidly switched from air to nitrogen. The appropriate boundary conditions are constant DO concentration at the PDMS aeration membrane/media interface and a zero flux boundary condition at the microbioreactor’s base. The initial condition for DO concentration is a constant at the value in equilibrium with air. i.e.

$$C\left(0,t\right)=C_{N_{2}}^{*}=0$$

$$\frac{\partial C}{\partial z}\left(L,t\right)=0$$

$$C(z,0)=C_{air}^{*}$$

Using separation of variables and a Fourier series the following solution can be prepared.

$$C(z,t)=\sum_{n=1}^{\infty} \frac{2C_{air}^{*}}{\lambda_{n}L}\sin\left(\lambda_{n}z\right)e^{-D\lambda_{n}^{2}t}$$

Where $\lambda_{n}=\frac{\pi\left(2n-1\right)}{2L}\Rightarrow\lambda_{n}^{2}=\frac{\pi^{2}\left(2n-1\right)^{2}}{4L^{2}}$

From above, the lowest eigenvalues can be calculated.

$$\lambda_{1}^{2}=\frac{\pi^{2}}{4L^{2}},\text{ }\lambda_{2}^{2}=9\frac{\pi^{2}}{4L^{2}},\text{ }\lambda_{3}^{2}=25\frac{\pi^{2}}{4L^{2}},\text{ ...}$$

Accordingly, the 1^st^ eigenmode dominates the decay spectrum, and hence the fitted k_L_a value. From equation this gives the following values for OUR_max_ and k_L_a.

$OUR_{\max}=\left(\frac{\pi}{2}\right)^{2}\frac{D}{L^{2}}C^{*}\Rightarrow\left.k_{L}a\right|_{dg}=\left(\frac{\pi}{2}\right)^{2}\frac{D}{L^{2}}$,

This approximation of the dynamic gassing-out method gives fairly close correspondence with the OUR_max_ and k_L_a values calculated for a diffusion reactor at steady state with constant OUR. i.e. $\left.k_{L}a\right|_{dg}\approx\left.1.23k_{L}a\right|_{ss}$.

This analysis assumed that OUR was homogenous within the reaction medium. If cells settle out, this will create a situation where most of the oxygen is consumed near the bottom of the reactor.

If the cells are considered as a homogeneous layer at the bottom of the reactor, and their consumption is given by the flux at the bottom of the reactor, then OUR_max_ at steady state will again coincide with zero oxygen concentration at the bottom of the reactor. Solving the diffusion equation with $OUR\left(z\right)=0$ and boundary conditions of $C(0,t)=C^{*}$ and $C(L,t)=0$. gives the following solution for DO concentration:

$C\left(z\right)=C^{*}\frac{(L-z)}{L}$.

The flux at the bottom of the reactor is given by $C^{*}\frac{D}{L}$, so after normalizing for volume *OUR*_max_ is given as follows:

$$\left.OUR_{\max}\right|_{ss}=C^{*}\frac{D}{L^{2}}$$

i.e. *k_L_a* is given by:

$$\left.k_{L}a\right|_{ss}=\frac{D}{L^{2}}$$

Hence a correction factor of ~ 0.41 may be required when correlating these reactors’ steady state performances to those predicted from dynamic gassing-out results.

Finally, there is the case where all cells have settled to the bottom of the bioreactor, but it remains well mixed. Oxygen consumption is again modelled by a point sink at the bottom of the bioreactor, but the well mixed media means that DO concentration is approximately constant and equation should remain a valid model. Hence the OUR_max_ and k_L_a values are calculated as for a well-mixed bioreactor.

**REFERENCES**

Lee HLT, Boccazzi P, Ram RJ, Sinskey AJ. 2006. Microbioreactor arrays with integrated mixers and fluid injectors for high-throughput experimentation with pH and dissolved oxygen control. Lab on a Chip 6(9):1229-1235.
